# Vienna Letter

**Published:** 1882-07

**Authors:** W. T. Belfield

**Affiliations:** Vienna


### Foreign Correspondence.

ARTICLE VIII.

VIENNA, JUNE 3, 1882.

EDITORS MEDICAL JOURNAL AND EXAMINER:

The iodoform strife seems to have subsided; an occasional grumble is still heard—Czerny of Heidelberg being the latest to put in a mild protest against the general substitution of iodoform for carbolic acid in surgical dressings. The discussion will have one eminently beneficial result—not by any means the exclusion of the agent from a very prominent place in the surgeon's armament, but simply the inculcation of proper caution in its use. That iodoform is not perfectly innocuous, as was at first supposed, is now evident; but neither is carbolic acid; and the valuable properties of the one are as unquestionable as those of the other. At any rate the Vienna surgeons, from Billroth down, are calmly using iodoform as the general dressing, and show no disposition to "weaken" under the furious onslaughts of Kocher *et al.* One fact, however, seems to be generally admitted, that iodoform does not afford the same protection against erysipelas as carbolic acid; hence surgeons may be compelled to resort temporarily at least to the acid, especially in hospital practice.

Koch's tubercle bacilli are the object of general attention at present; they are unquestionably to be seen in every case of tuberculosis—if one has a homogeneous (oil) immersion lens, considerable experience in histological manipulation, and patience. Several methods have been already devised for bringing their detection in sputum within the reach of the practitioner, with the hope that the microscope may afford a definite diagnosis long before the physical changes are such as can be detected by the stethoscope. Ehrlich, Frerich's assistant in Berlin, has devised a plan by which he says the bacilli can be seen through a good water immersion lens giving an amplification of 5-600 diameters. I have not yet used Ehrlich's methed, but I know by experience that Koch's is too exacting to possess much value for clinical purposes. Moreover it remains to be seen whether the bacilli can be detected in sputum with sufficient certainty to establish a diagnosis, before the usual symptoms and signs are present.

Neisser, in Breslau, who in 1879 published the discovery of a micrococcus which was to be found in every case of specific blenorrhœa either of urethra or of eye, but nowhere else, has recently recorded his conviction that this "gonococcus," as he christens it, is the specific agent in producing the disease; and quotes Aufrecht, Ehrlich and Gaffkey, the ophthalmologists Leber, Sattler and Hirschberg in support of his statements. But until recently the *experimentum crucis*—successful inoculation of the micrococci—had never been performed from the simple fact that animals are not susceptible to the gonorrhœal contagion, and human subjects have always preferred to acquire theirs in another way. Recently, however, Bokai, in Pesth, was so fortunate as to find six philanthropic students who placed their urethras at the disposal of science. With the "gonococci" which Bokai had cultivated artificially from gonorrhœal discharge, the six were inoculated; and three had the satisfaction of exhibiting a week later a classical gonorrhœa. For one somewhat familiar with the natural history of medical students, the experiment would have been far more convincing if the dauntless three had been kept in solitary confinement for a week before and after the inoculation. That these organisms are present in specific urethritis and conjunctivtis, and that they are not to be seen in pus from other sources, I have myself observed in numerous cases; but however great the probability as to the relation of cause and effect may be, the proof, as Neisser himself admits, is still lacking.

Still another bacterial disease has been the subject of recent experiment—this time on a large scale. Pasteur's assertion that animals can be protected by vaccination against splenic fever, which in Europe destroys immense numbers of cattle, has been recently subjected to a practical test by the Prussian government. Your readers may remember that a similar experiment was made last fall by the Hungarian government, which placed at Pasteur's disposal several hundred sheep and cattle, half of which were vaccinated by Pasteur's assistant Thuillier, while the other half were left unprotected. About thirty days later all were inoculated with splenic fever bacillus which had been cultivated in Paris by Pasteur. The results were not entirely satisfactory: (1) Because several animals succumbed to the protective vaccination: (2) Because several others of the vaccinated died of intercurrent diseases during the vaccination period, while the non-vaccinated animals remained without exception healthy until finally inoculated. The government committee concluded from this fact that in consequence of the vaccination the ability of the animal to combat pathological influences was materially diminished: (3) Because several vaccinated animals yielded as rapidly to the poison as the unvaccinated. At the close, 14.5 per cent. of the vaccinated and 94 per cent. of the unvaccinated animals were dead. The committee reported advising, not simply that the government withhold its official sanction, but even that the vaccination by private individuals should be absolutely forbidden.

The results recently attained in Prussia are decidedly more favorable. With material cultivated in Paris, six cows and twenty five sheep were vaccinated by Pasteur's assistant, April 5th, and again Apr. 19—an equal number of animals being reserved for comparison. Three sheep died of splenic fever after the second vaccination; several others had slight fever. On May 6th all (twelve cows, forty seven sheep) were subcutaneously inoculated with the blood of a sheep which had died of splenic fever thirty hours previously—each sheep receiving one, each cow two and a half decigrammes of the blood. Three days later twenty-four sheep and three cows (all unvaccinated) were dead, the others sick; while all the vaccinated animals were perfectly well. Examination of several of the dead animals (by Virchow among others) proved the presence of the characteristic bacilli in the blood.

Pasteur ascribes the death of the three sheep after vaccination to a peculiarity of race and promises to send a "vaccin adapté" for such cases.

The decision of the government in the matter has not yet been made public.

W. T. BELFIELD.

